# Addressing Social Context in Health Provider and Senior Communication Training: What Can We Learn From Communication Accommodation Theory?

**DOI:** 10.7759/cureus.12247

**Published:** 2020-12-23

**Authors:** Beheshta Momand, Adam Dubrowski

**Affiliations:** 1 Health Sciences, Ontario Tech University, Oshawa, CAN

**Keywords:** primary health care, communication simulation, communication in primary health care, seniors, elderly population, communication accommodation theory

## Abstract

The ability to communicate enables people to share information, thoughts, and concerns with others in a certain time and place. Communication plays a fundamental role across a variety of institutions. However, the stakes are exceptionally high in primary health care (PHC). Poor communication in PHC increases the patient's risk of medication errors, patient injury, delay in treatment, and/or death. Effective communication is especially critical when health providers communicate with seniors because aging is partly responsible for physical, mental, and social/emotional changes. Studies have suggested that simulations are an effective means to train health providers in the development/enhanced communication skills; however, current educational programs focus on physical and cognitive aspects of aging. This editorial highlights possible contributions from the communication accommodation theory (CAT) to structure a communication training strategy that may help to improve healthcare providers’ ability to converse and connect with the vulnerable older population and address their social and emotional well-being.

## Editorial

The base foundation in the delivery of optimal healthcare services to the public is through effective communication - this includes communication with patients and between health providers. Effective communication in health care is vital when discussing patients’ medical history, procedures and addressing patients’ needs, medications, and treatments. When health providers lack communication skills, it may result in vital information not being conveyed appropriately and effectively, which increases the patient’s risk of injuries and malpractice [1]. According to the Canada Survey of Canadian healthcare facilities, nearly 25% of health providers have specified communication as a leading cause of documentation errors impacting the safety of patients [1].

The need for effective communication can impact the provision of optimal care for individuals of all ages. However, seniors (65+) are a subgroup of the general population that is prone to health conditions that can affect their physical, mental, and/or social/emotional well-being. Therefore, it becomes imperative for health providers to have effective communication skills prior to engaging with elderly patients. However, current literature suggests that the health personnel-senior communication is not very effective, mainly as the result of a lack of education which can make it difficult for health providers to know when to ask probing questions, how to address patients’ needs and concerns, and how to effectively deliver key information [2].

Age-related changes

Changes Seen in Elderly Persons Between 65 and 79 Years of Age

Several changes can occur during this phase of one’s life. The changes can be categorized into three domains: (1) physical, (2) mental, and (3) social/emotional health. In the physical aspect, an elderly individual's body naturally declines in some senses and physical abilities. Examples of physical changes that can occur include but are not limited to hearing loss, skin changes, heart troubles, vision loss, decrease in bones and joint function, teeth decline, and more. In the mental health aspect complications that can occur include but are not limited to a decline in memory skills, isolation, anxiety disorders, dementia, and more. In the social/emotional aspect, individuals try to balance independence with the need to be dependent in some ways. Examples of changes that occur are friends begin to die, feeling of loneliness, and memory [3].

Changes Seen in Elderly Individuals 85 Years and Older

During this stage of an elderly individual’s life, the physical component begins to decline at a higher rate. Physical changes include an increased risk for chronic diseases, bones and muscles shrink in size and flexibility, dry and brittle skin, hearing impairments, and the body's ability to regulate heat decreases. During the mental health component, an individual’s memory and speed for skills decline, confusion is often displayed, and speed of learning declines; individuals may have a hard time understanding information, which may interfere with adhering to medication and treatment regimes. During the social/emotional aspect, individuals continue to live as independently as possible, accept changes that arise with aging, and come to terms with the end of life and personal losses [3].

What is the current gap in health provider-senior communication training?

One effective way to enhance communication education training for health providers who work with seniors is through simulation. Simulation is a technique used for learning and practice that allows individuals to amplify/replace real-life experiences with guided ones. Studies have suggested that ineffective communication in healthcare could be addressed through simulation [4]; the current literature addresses physical and cognitive areas of aging, rather than the social aspect of communication and aging. For example, the Canadian Association of Schools of Nursing (CASN) framework was developed to educate nursing students about the core competencies they should possess in gerontological nursing as it relates to older persons and their families. As comprehensive as it may be, the framework does not adequately address the social aspects of an elderly individual [3]. The social aspect encompasses the process of caring for patients who require more than just the treatment for a disease; it requires health providers to take a holistic approach; to understand the patient's physical, cognitive, and social/emotional needs; and to communicate effectively.

Proposed solution

To date, there are no known theory-based models for simulation-based training for healthcare providers focused on the social and emotional aspect of communication with the elderly. One theoretical framework that can be used to understand and potentially improve communication is the communication accommodation theory (CAT), which emphasizes the minimization of social differences in people’s communication [5]. This can be done by matching your vocabulary, accent, and cadence with others. In general, the CAT is based on two assumptions. First is that people make behavioral changes to attune their communication to their communication partner. The second is that the effectiveness of communication is directly related to the extent to which people perceive their communication partner as appropriately attuning to them. Therefore, according to CAT and these two assumptions, people adjust (or accommodate) their communication styles to others involved in the communication and the larger context in which the communication occurs. There are several advantages to this: Message sender gains approval from the receiver; there is an overall increase in the efficiency of communication; and the sender maintains a positive social identity. Thus, in general, CAT links the language, context, and social identity of the parties involved in communication. Furthermore, CAT is focused not only on how the message sender and the message receiver interact during the communication process but also on how the communicator accommodates to a larger societal context.

Moreover, the accommodation process is also divided into two categories according to the CAT theory. One is convergence, which looks at how individuals naturally adapt their communication to decrease social differences, while the other one is divergence, which is the process of non-adaptation of communication. The accommodation process has been further divided into two categories called convergence and divergence. To further explain the two categories, we present a case of an 80-year-old patient who suffers from Alzheimer’s disease. When communicating with this patient a health professional case uses the convergence method by speaking slowly and clearly, maintaining eye contact, giving the patient time to respond, being patient, offering reassurance, and asking one question at a time, thus adapting to the patient's communication methods. While in the divergence method the health professional would show disapproval of the patient by talking too fast, overwhelm them with information, and not giving them enough time to respond.

Therefore, one can suggest that CAT has the potential to be embedded within simulation-based training to address and accommodate the cultural needs of the patient population. For example, CAT recommends that individuals alter their communication to non-verbal means such as gestures. By sending patients non-verbal cues such as head nodding or eye contact, it can demonstrate empathy, understanding, and acknowledgment. Moreover, health providers can also eliminate social gaps by understanding a senior patient's environment. This can be done by taking a scan of the individual’s home and looking for things that they like such as paintings, pictures, gardening, and/or cooking. Understanding the personal environment can assist a health provider to establish trust, promote communication, increase patient medication adherence, and identify potential barriers to accessing care.

Conclusion

Effective communication is an important part of the healthcare process. It allows for the exchange of ideas, thoughts, and concerns to be delivered via verbal and non-verbal means. Without effective communication, the incidence of patient injuries, medical errors, and death may increase. Compared to other age groups, older adults are at a higher risk of experiencing ineffective communication when receiving care. This puts seniors at a greater risk related to physical and cognitive aspects of aging, such as forming complications or exacerbating existing ones causing serious harm. As previously mentioned, poor communication can also affect a senior's physical, emotional, and social well-being. This can result in seniors feeling despair and isolated. Studies have suggested that simulations are a great way of training health providers in developing effective communication skills. However, current training programs focus on the physical and cognitive aspects of aging. We propose that using CAT to structure a communications training strategy may help to improve health providers’ ability to converse and connect with this vulnerable population and address their social and emotional well-being.

## References

[1] Guerriere Guerriere, M M (2020). Strong foundations: tackling healthcare’s failure to communicate. Telus Health.

[2] Kee JWY, Khoo HS, Lim I, Koh MYH (2018). Communication skills in patient-doctor interactions: learning from patient complaints. Health Professions Education.

[3] Martin Martin, Guse Guse, Baker Baker (2020). Entry‐to‐practice gerontological care competencies for baccalaureate programs in nursing. https://www.casn.ca/wp-content/uploads/2016/09/FINAL_CASN-GERONTOLOGY-COMPETENCIES-FINAL.pdf.

[4] Luctkar-Flude M, Baker C, Pulling CA, McGraw R, Dagnone D, Medves J, Turner-Kelly C (2010). Evaluating an undergraduate interprofessional simulation-based educational module: communication, teamwork, and confidence performing cardiac resuscitation skills. Adv Med Educ Prac.

[5] Giles Giles, H H (2016). Communication Accommodation Theory.

